# Supplementary material for: Analysis of the distraction impact on driving performance across driving styles: A driving simulator study in various speed conditions
Source: PLoS One. 2025 Dec 4;20(12):e0336480. doi: 10.1371/journal.pone.0336480 (PMC12677451; doi:10.1371/journal.pone.0336480)
Supplement: Attachment — Submitted filename: rebuttal_letter.pdf [file pone.0336480.s002.pdf]

# Authors' response

# PONE-D-25-19007

# Analysis of the distraction impact on driving performance across driving styles: A driving simulator study in various speed conditions

Mobina Faqani, Habibollah Nassiri, Mahdi Rezaei, Mohsen Ramezani

The authors sincerely thank the editor for managing the review process and the reviewers for their thorough and constructive feedback. In response, we have carefully revised the manuscript to improve its clarity, rigor, and overall scientific value. Specifically, we have added more detailed information on participant characteristics, clarified the experimental design and discussed its limitations, improved the description of the distraction tasks, expanded the justification for the use of K-means clustering and clarified the criteria for driving style classification, revised the introduction, and enhanced the discussion section. All revisions are clearly marked in blue in the revised manuscript.

**Note:** The figure and table numbers referenced in the reviewers' comments pertain to the original version, while those in our responses refer to the revised manuscript. Please find our detailed, point-by-point responses to each reviewer comment below.

*Reply to Reviewer #1*

**C1.0** This paper focuses on a interesting and meaningful topic, where the intersection between driving style and driving performance under MPDD conditions were investigated. However, there are several issues to be clarified.

**R1.0** We thank the reviewer for the thoughtful and encouraging feedback, as well as for recognizing the importance and relevance of our study. In response, we have carefully considered each point raised and made the corresponding revisions to the manuscript, as detailed below.

---

**C1.1** Page 4, Lines 96-100. It would be better to describe more information of participants, such as the accident experience, vision and hearing conditions.

**R1.1** We appreciate the reviewer's suggestion regarding the inclusion of more detailed participant information. We have revised the manuscript accordingly and included additional details:

"A total of 40 neurologically healthy participants with normal vision and hearing abilities, all licensed drivers, were recruited for this study, comprising 7 females and 33 males. The participants' ages ranged from 18 to 42 years (M = 28.68, SD = 6.9), with driving experience ranging from 1 to 22 years (M = 9.1, SD = 6.44). Of the sample, 55% reported using a mobile phone while driving more than three times per week, indicating a relatively high prevalence of mobile phone use in their driving routines. Additionally, 10% of participants reported having been involved in a road accident while using a mobile phone. In a pre-survey, participants rated the potential for mobile phone use to cause distraction and accidents at an average of 3.18 out of 4, indicating a strong awareness of the associated risks."

---

**C1.2** Page 4, Line 101. The authors adopted randomized order to minimize potential learning effects. Although the randomized order is valid to some extent, the use of a Latin-square experimental design is more standardized and effective.

**R1.2** Thank you for this helpful suggestion. Although the present study used simple randomization to counterbalance potential learning effects, we agree that a Latin-square design would provide a more systematic and statistically efficient solution. We have therefore acknowledged this limitation in the revised manuscript:

"Future research should consider a larger and more diverse sample, incorporating additional behavioral patterns and broader driving scenarios to enhance the generalizability of the findings. Task order was counterbalanced through random assignment to mitigate learning effects; however, future studies should employ a Latin-square experimental design, which offers a more rigorous and standardized means of controlling sequence effects."

---

**C1.3** Page 4, Line 106. The driving simulator shown in Fig 1 does not appear to have motion simulation capabilities and is therefore not a high-fidelity driving simulator. In the field of driving behavior analysis, driving simulators that only have visual and auditory simulations are usually referred to as medium-fidelity driving simulators.

**R1.3** Thank you for bringing this to our attention. We have revised the terminology accordingly and now refer to the simulator as a "medium-fidelity" driving simulator in the revised manuscript. Additionally, we have acknowledged this limitation in the discussion of the study's limitations and future research directions.

"The experiments were conducted using a medium-fidelity driving simulator at the Road Traffic Injury Research Center in Tabriz, Iran."

"While this study utilized a medium-fidelity driving simulator, the use of high-fidelity simulators in future work may enhance validity."

---

**C1.4** Page 5, Line 132-133. The instructions for the participants need to be further clarified. For example, the participants were required to follow the lead vehicle and were not allowed to overtake the vehicle or change lanes under any circumstances.

**R1.4** Thank you for highlighting this point. While our original text mentioned the car-following setup and overtaking restriction ("During the experimental trials, participants were instructed to drive according to their natural driving habits, with a specific emphasis on avoiding overtaking the lead vehicle"), we agree that the instructions could be stated more clearly. We have revised the manuscript to clarify the participants' driving instructions, explicitly mentioning both the requirement to follow the lead vehicle and the prohibition of overtaking or changing lanes:

"During the experimental trials, participants were instructed to drive according to their natural driving habits, while consistently following the lead vehicle and strictly avoiding any overtaking or lane changes under any circumstances."

---

**C1.5** Page 6, Table 1. The initial case form of the variables in the table is not standardized.

**R1.5** Thank you for pointing this out. We have revised Table 1 to ensure consistent and standardized formatting of all variable names, using uniform initial capitalization throughout.

Table 1: Driving Variables recorded from the Simulator

| Variable | Unit |
|---|---|
| Speed | $m/s$ |
| Relative speed | $m/s$ |
| Space headway | $m$ |
| Longitudinal acceleration | $m/s^2$ |
| Degree of acceleration pedal | $\circ$ |
| Degree of brake pedal | $\circ$ |
| Steering wheel angle | $\circ$ |
| Lane deviation | $m$ |

---

**C1.6** Page 7, Lines 187-193. Why the authors only adopt conditions (i) to (iv) to recognize participants driving style? There were six condition in total.

**R1.6** Thank you for this insightful question. There were three main reasons for focusing on conditions (i) to (iv) when identifying participants' driving styles.

First, our goal was to assess participants' inherent driving behavior—particularly voluntary decisions such as headway maintenance, throttle, braking, and steering inputs—without strong external constraints. Conditions (i)–(iv) reflect steady-state car-following scenarios across a range of speeds, from free-flowing to mildly congested traffic, which are well-suited for revealing individual driving tendencies. In contrast, conditions (v) and (vi) introduced highly reactive situations, including stop-and-go queues and lead-vehicle cut-ins. These scenarios elicit context-driven responses that tend to obscure individual driving style.

Second, our analysis showed that using clustering variables (e.g., Max-S, Max-ABS-LA, Min-SH) under conditions (v) and (vi) did not yield statistically distinguishable clusters, further limiting their effectiveness for style classification.

Finally, conditions (v) and (vi) were specifically designed as performance testbeds for evaluating the effects of mobile phone distracted driving (MPDD). To avoid circular reasoning, we deliberately excluded these conditions from the style classification process and reserved them solely for assessing MPDD impacts.

We have clarified this rationale in the revised manuscript to better reflect the methodological decisions.

"Our assumption was that participants exhibited one of two distinct driving styles—aggressive or non-aggressive—under conditions (i) to (iv) in the baseline experiment. Conditions (i)–(iv) were used to distinguish aggressive from non-aggressive drivers because they represent steady-state car-following in free-flow or mildly congested traffic, where headway choice, acceleration, braking and steering are largely voluntary. In contrast, conditions (v) and (vi)—stop-and-go queues and lead-vehicle cut-ins—provoke highly reactive responses that obscure underlying style differences; clustering metrics such as Max-S, Max-ABS-LA and Min-SH did not form distinct groups in these conditions. We therefore excluded conditions (v) and (vi) from style classification and reserved them as performance testbeds for assessing the impact of MPDD, avoiding circular reasoning."

*Reply to Reviewer #2*

**C2.0** Although the topic of this manuscript holds certain practical significance and the authors have made efforts in reviewing the relevant literature, there remain notable shortcomings. Specifically, the research objectives and innovative contributions are not clearly articulated; the experimental design suffers from potential confounding factors and lacks sufficient detail; and the definition and interpretation of key variables are not rigorous.

**R2.0** We sincerely thank the reviewer for their thoughtful and constructive feedback. We acknowledge the concerns raised regarding the clarity of the research objectives and contributions, the experimental design, and the definition and interpretation of key variables. In response, we have thoroughly revised the manuscript to enhance the articulation of our research aims and innovative aspects, clarified the experimental setup to address potential confounding factors, and refined the definitions and explanations of key variables to ensure greater rigor and precision.

We address each of the reviewer's specific comments in detail below and hope that these revisions improve the quality and clarity of the manuscript to the reviewer's satisfaction.

---

**C2.1** In the Introduction, the authors state that mobile phone-related distracted driving (MPDD) has become a key concern in road traffic safety. However, the cited references [6]–[12] mainly document the co-occurrence of distracted driving and MPDD across different countries, without providing concrete evidence on how MPDD specifically increases crash risk. It is recommended that more targeted literature be included to clarify the specific mechanisms through which MPDD impacts driving safety.

**R2.1** We thank the reviewer for this valuable suggestion. We have now added a dedicated sentences that synthesizes recent naturalistic-driving studies, fatal-crash analyses and meta-analyses demonstrating how mobile-phone use elevates crash and near-crash risk. These studies show that visual-manual tasks (e.g., texting, dialing) double to quintuple crash odds, largely through increased eyes-off-road time, impaired hazard detection and slowed reaction times. The revised text cites new references. We believe this addition clarifies the safety mechanisms of MPDD and directly addresses the reviewer's concern.

"Mobile phone-distracted driving (MPDD) has emerged as a critical area of concern in road safety, with substantial evidence pointing to its role in increasing the risk of traffic incidents Caird et al. (2018); Organization (2015); Zhu et al. (2018); Peng et al. (2023); Okati-Aliabad et al. (2024). In 2016, distraction contributed to 9% of all fatal crashes, underscoring its detrimental impact on safety National Highway Traffic Safety Administration (NHTSA) (2019). Drivers who read or compose text messages exhibit slower stimulus-response times, lower speeds, poorer lane tracking, shorter headways, and fewer forward glances—factors that together heighten the likelihood of safety-critical events compared with undistracted driving Basacik et al. (2011); Caird et al. (2014); Choudhary and Velaga (2017). One study reported that phone use lengthened reaction time by roughly 30% and markedly impaired the driver's ability to keep a safe gap to the lead vehicle Basacik et al. (2011). Crash-risk estimates range from two- to nine-fold higher for drivers engaged in mobile-phone tasks than for those who are not distracted McEvoy et al. (2005); Klauer et al. (2014). Data from the Second Strategic Highway Research Program Naturalistic Driving Study (SHRP 2) further show that hand-based phone activities raise crash odds ratios substantially; texting while driving, for instance, increases risk by a factor of about 6.1 Dingus et al. (2016); Antin et al. (2019). The study conducted indicated that there is a relationship between the use of cellular telephones while driving and a subsequent crash with fatalities. The researchers found out that the use of phone for texts and Internet to be positively associated with road crash fatalities with incremental risks of 8.4%, and 54.6%, respectivelyGariazzo et al. (2018). Similarly, Perez et al. (2024) concluded that distractions

from smartphone apps and social media—combined with reduced situational awareness and risk perception—may significantly elevate crash risk Perez et al. (2024).

A sizeable literature also documents how widespread this behaviour is across regions and demographic groups. For instance, research conducted in China found that 84.1% of surveyed drivers (from a sample size of 414) reported engaging in phone conversations at least weekly while driving Shi et al. (2016)."

---

**C2.2** Despite conducting a relatively comprehensive review of existing studies, the Introduction fails to extract the core research question and clearly define the study's objectives. This lack of focus obscures the novelty and significance of the research. The authors are advised to better articulate the research motivation and explicitly state the scientific problem this study aims to address, along with its potential contributions.

**R2.2** We appreciate the reviewer's insightful comment. In response, we have included a dedicated paragraph to better highlight the novelty and significance of the study.

"While previous studies have examined either driving style classification or the effects of mobile phone distraction on driving behavior independently, the combined influence of mobile phone distraction on drivers with varying driving styles remains largely underexplored. This study aims to fill this gap by addressing the following research question: How does mobile phone distraction—specifically hands-free (HF) conversations, which primarily involve cognitive distraction, and texting, which primarily involves manual distraction—affect driving performance, including lateral maneuvering across different traffic conditions, Acceleration Reaction Time (ART), and Time to Initial Braking Location (TIBL), both with and without accounting for distinct driving styles (aggressive, moderate, and conservative)?

Aggressive driving typically involves higher speeds, sudden accelerations, abrupt steering, and sharp maneuvers, while conservative driving is characterized by smoother maneuvers and more gradual acceleration and deceleration. A moderate driving style, as the term suggests, sits between these two extremes Mohammadnazar et al. (2021). Previous studies have categorized drivers based on behavioral observations and driving metrics, such as speed variance, acceleration distribution, and lane deviation Doshi and Trivedi (2010); Higgs and Abbas (2014); Qi et al. (2015), or through surveys that measure self-reported driving behaviors Eboli et al. (2017); French et al. (1993); Ishibashi et al. (2007). For this study, driving style classification considers speed, acceleration, and distance to the lead vehicle. Given our research objectives—to examine the effects of mobile phone distraction, to categorize drivers into distinct driving styles, and to compare performance outcomes with and without considering driving style—the study is designed around a car-following task. Participants follow a lead vehicle through six different traffic conditions: free-flow, coherent-moving flow, synchronized flow, traffic jam, recovery from traffic jam, and collision avoidance. We hypothesize that traffic congestion and the relative position of the lead vehicle Emo et al. (2016); Li et al. (2020a,b); Stephens and Groeger (2014) significantly affect driving aggression, making this scenario suitable for exploring the interaction between driving style and distraction type."

---

**C2.3** The experiment involves six types of traffic conditions, yet each condition lasts only for a short period, and the vehicle's state changes continuously. This setup may introduce interference effects that compromise the drivers' ability to perform the tasks as intended, thereby undermining the reliability of the collected data. The experimental design should be refined, or the strategies used to mitigate such interference should be clarified. Additionally, Figure 2 lacks necessary details; it is recommended to include the time–speed profiles at key event points to improve clarity.

**R2.3** We appreciate the reviewer's suggestion, which helped us strengthen the clarity and robustness of the experimental design and its presentation. We have acknowledged the potential for interference

effects and now discuss our control strategies more explicitly. These effects are, in fact, inherent to our experimental framework, which was adapted from the scenario proposed by Yan et al Yang et al. (2018). Our intent was to simulate realistic driving conditions in which drivers must respond to continuous and variable traffic demands. This setup allows us to both identify underlying driving styles and assess performance under distraction. These elements were essential to replicating real-world driving challenges and evaluating behavior in a naturalistic yet controlled environment.

As suggested, we have revised the paragraph describing Fig 2 to include key event points for each condition. This enhancement provides a clearer visualization of the scenario timeline and helps clarify the structure of the experimental sequence.

"The total driving duration of 574.931 seconds was segmented into six distinct traffic conditions: 19.45 to 64.5 seconds for the first condition, 67.288 to 231.175 seconds for the second, 236.731 to 414.5 seconds for the third, 417.285 to 473.961 seconds for the fourth, 496.733 to 516.19 seconds for the fifth, and 566.171 to 574.931 seconds for the sixth. The speed profile of the lead vehicle under different traffic conditions is illustrated in Fig. 2."

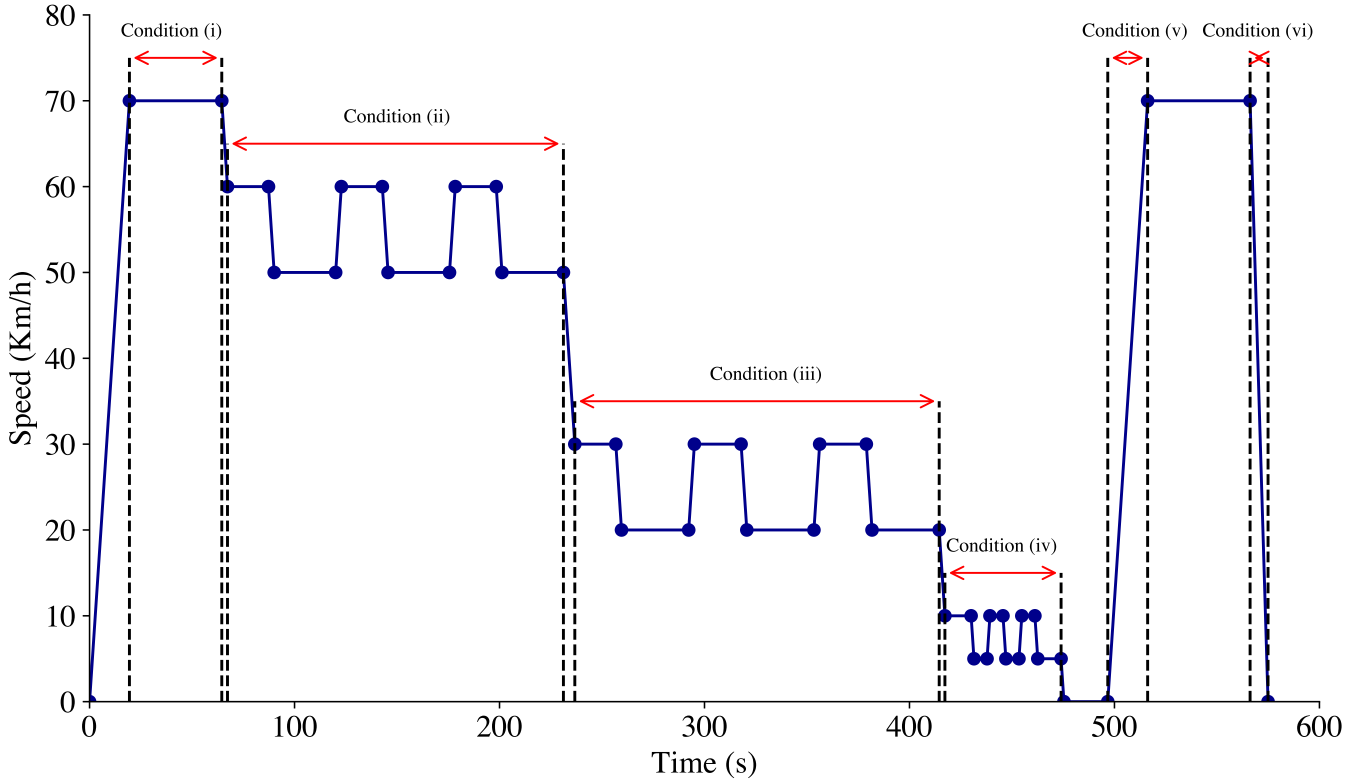


Figure 2: Lead vehicle speed profile across different traffic conditions: (i) free-flow; (ii) coherent moving flow; (iii) synchronized flow; (iv) jam density; (v) recovery from jam density; (vi) collision avoidance.

---

**C2.4** The current description of the mobile phone task is insufficient to evaluate whether it effectively induces cognitive distraction. Detailed information should be provided on the experimental procedures, such as how distraction tasks are triggered, the content and duration of phone calls, the type and demands of text messaging tasks, etc. Moreover, the method for quantifying cognitive load should be specified, and evidence should be presented to confirm that the distraction task significantly increases cognitive demand on the driver.

**R2.4** We thank the reviewer for this thoughtful comment and the opportunity to clarify the design and implementation of the mobile phone distraction tasks. As mentioned in the original manuscript,

the cognitive distraction task involved participants listening to complete arithmetic questions and providing verbal responses. In the revised version, we have expanded this section to offer a more detailed explanation of the task structure, delivery, and timing:

"In the hands-free conversation task, participants listened to complete arithmetic questions and were required to provide appropriate verbal responses. The arithmetic questions involved calculating the sum of two-digit numbers presented in a random difficulty order. For instance, the sum 67+89 = 156 is considered more challenging, while 10+20 = 30 is regarded as a simpler calculation.

In the texting task, participants received the same arithmetic questions and were required to both compute and text their answers. Similar to the conversation task, the arithmetic problems involved summing two-digit numbers with varying difficulty levels.

Both the hands-free calling and texting tasks were initiated precisely at the beginning of the respective experimental conditions and continued until the end of the scenario, ensuring consistent exposure to cognitive load throughout the task duration."

To assess the effectiveness of these tasks in inducing cognitive distraction, we administered a post-experiment survey asking participants to rate the level of distraction experienced during each task. On average, participants rated the hands-free task at 3.7/5 and the texting task at 4.2/5, indicating that both were perceived as cognitively demanding, with texting being more intrusive. Such subjective ratings are well-aligned with established distraction research: for instance, Strayer et al. (2015) employed a cognitive distraction scale—including subjective (NASA-TLX) assessments—across in-vehicle tasks ranging from low-demand (radio listening) to high-demand (hands-free and handheld phone conversation), demonstrating clear increases in perceived workload that correlated with driving impairments Strayer et al. (2015). We believe these clarifications provide a more comprehensive understanding of our distraction protocol and its impact on driver cognitive load.

---

**C2.5** The study classifies participants into two driving style groups. However, the criteria for this classification are unclear, and the rationale for choosing the K-means clustering method has not been justified. The authors should explain why K-means is suitable for this context and clarify its methodological advantages in this study.

**R2.5** We sincerely thank the reviewer for this insightful comment and the opportunity to clarify both the classification criteria for driving styles and the rationale for selecting the K-means clustering algorithm.

K-means clustering was chosen due to its simplicity, interpretability, and effectiveness in identifying natural groupings within behavioral datasets—particularly when the goal is to explore latent patterns in a continuous feature space. Given the exploratory nature of our study and the relatively moderate sample size, K-means offered a transparent and computationally efficient method well-suited to our context.

The clustering was performed based on key behavioral indicators extracted from baseline driving conditions, including maximum speed (Max-S), maximum absolute lateral acceleration (Max-ABS-LA), and minimum headway (Min-SH). These variables were selected as they reflect fundamental aspects of driving behavior—speed preference, maneuvering intensity, and following distance—which are widely recognized as core indicators of driving style.

To ensure methodological robustness, we tested alternative clustering techniques such as hierarchical clustering (with and without PCA) and DBSCAN. However, these methods either resulted in imbalanced groupings, lower silhouette scores, or poor separability. In contrast, K-means with $k = 2$ consistently produced more stable and interpretable clusters.

The assumption of two clusters was theoretically motivated by the hypothesis that drivers tend to fall into one of two broad behavioral profiles—typically referred to as "conservative" and "aggressive"

styles. This choice was further supported by internal validation metrics, including the elbow method, silhouette analysis, and the gap statistic, all of which indicated that $k = 2$ was an appropriate and data-driven choice.

The manuscript primarily presents the final clustering results to maintain clarity and conciseness. Nonetheless, in response to the reviewer's helpful comment, we have added brief but essential clarifications to address the concern while preserving the overall focus of the section.

"Our assumption was that participants exhibited one of two distinct driving styles—aggressive or non-aggressive—under conditions (i) to (iv) in the baseline experiment. The assumption of two clusters was theoretically motivated by the hypothesis that drivers tend to fall into one of two broad behavioral profiles—typically referred to as "conservative" and "aggressive" styles. This choice was further supported by internal validation metrics, including the elbow method, silhouette analysis, and the gap statistic, all of which indicated that $k = 2$ was an appropriate and data-driven choice."

"Accordingly, K-means clustering was employed separately for each of these conditions to classify participants into aggressive or non-aggressive driving styles. To ensure methodological robustness, we tested alternative clustering techniques such as hierarchical clustering (with and without PCA) and DBSCAN. However, these methods either resulted in imbalanced groupings, lower silhouette scores, or poor separability. In contrast, K-means with $k = 2$ consistently produced more stable and interpretable clusters. We explored different combinations of driving features across conditions (i) to (iv) to maximize the similarity in driving style classification. Additionally, we assessed cluster quality, stability, and interpretability, with a particular focus on the significance of K-means cluster centers."

---

**C2.6** The Discussion section is overly brief and mainly repeats the results without engaging in comparative analysis with existing literature. It is recommended to expand this section by contrasting the study's findings with prior research and analyzing possible reasons for any discrepancies, in order to highlight the scientific value and implications of the work.

**R2.6** We appreciate the reviewer's helpful observation. We have substantially expanded the Discussion to compare our findings with recent studies, (ii) explain convergent and divergent results. The new material appears of the revised manuscript.

" Our findings demonstrate clear, condition- and style-specific effects of MPDD on driving performance. Tables 10 and 11 summarize the influence of distraction types (hands-free vs. texting) on SDLP, ART, and TIBL across aggressive, moderate, and conservative drivers.

Table 10: Summary of the effect of MPDD on SDLP across all participants, and also participants categorized by driving styles. (“NS” (no significant), “+” (significant increase), and “-” (significant decrease).)

| | | HF conversation | | | Texting | | |
|---|---|---|---|---|---|---|---|
| | | Aggressive | Moderate | Conservative | Aggressive | Moderate | Conservative |
| SDLP | Free Flow | NS | | | + | | |
| | | NS | NS | NS | NS | + | + |
| | Coherent moving flow | − | | | + | | |
| | | NS | NS | − | NS | + | NS |
| | Synchronized flow | − | | | NS | | |
| | | NS | − | − | NS | NS | − |
| | Jam density | − | | | NS | | |
| | | NS | − | − | NS | NS | NS |

Table 11: Summary of the effect of MPDD on ART and TIBL across all participants, and also participants categorized by driving styles. (“NS” (no significant), “+” (significant increase), and “-” (significant decrease).

| | | HF conversation | | | Texting | | |
|---|---|---|---|---|---|---|---|
| | | Aggressive | Moderate | Conservative | Aggressive | Moderate | Conservative |
| ART | Recovery from jam density | − | | | + | | |
| | | NS | NS | − | NS | + | + |
| TIBL | Collision avoidance | NS | | | + | | |
| | | NS | NS | NS | NS | + | + |

Texting generally led to significant increases in SDLP across several traffic regimes, especially for moderate and conservative drivers under free-flow and coherent-moving flow conditions. Conversely, hands-free conversations had mixed effects: under jam density and synchronized flow, we observed a reduction in SDLP for conservative drivers. These results align with those of Giot et al. (2022) and Rumschlag et al. (2015), who reported that texting degrades lane-keeping more consistently than verbal distractions Giot et al. (2022); Rumschlag et al. (2015).

Interestingly, in specific low-speed, high-density situations, hands-free conversation appeared to stabilize lane control, possibly by increasing cognitive engagement without overloading visual-manual resources. This pattern supports the nuanced findings of Verster and Hartman (2015) and Caird et al. (2018), who suggested that hands-free interactions can sometimes enhance lane centering when drivers self-regulate speed and attention Hartman et al. (2015); Caird et al. (2018).

A particularly notable result emerged during the recovery from jam density phase. Here, texting significantly lengthened ART for moderate and conservative drivers, indicating impaired readiness to accelerate following congestion. However, hands-free conversation showed either negligible or decreased ART in this phase for some driver types, especially conservative drivers. This suggests a possible vigilance-enhancing effect of verbal interaction during low-demand traffic conditions.

These outcomes partially diverge from the aggregate findings of Caird et al. (2018), whose meta-analysis showed consistent reaction time impairments for all phone use types Caird et al. (2018). Our traffic-state-specific approach may account for this discrepancy by isolating traffic regimes where task demands interact differently with cognitive distraction.

TIBL during the collision avoidance phase was significantly impacted by texting across most driver categories, particularly moderate and conservative participants. While hands-free conversation had little effect on TIBL, the significant increases seen during texting align with prior studies showing degraded braking and hazard anticipation during visual-manual phone use Choudhary and Velaga (2019); Chouhan et al. (2025); Andrikopoulou and Spyropoulou (2025).

Our findings corroborate previous studies that identified texting as a major threat to lane-keeping and reaction time performance Yan et al. (2022); Austers et al. (2025). For instance, Yan et al. (2022) found a pronounced SDLP increase on straight segments during texting. Similarly, Chouhan et al. (2025) quantified texting as raising crash probability by over 27% due to reaction-time impairments. Moreover, studies like Ortega et al. (2021) and Andrikopoulou et al. (2025) demonstrated that mobile phone use worsens lateral deviation and reduces driver control, particularly among younger drivers or during multitasking events Ortega et al. (2021); Andrikopoulou and Spyropoulou (2025).

Driving style emerged as a critical moderating factor. moderate drivers exhibited the highest texting-related increases in SDLP and ART, likely due to inherently higher speed variability and less cautious behavior. On the other hand, conservative drivers showed more pronounced sensitivity to hands-free conversations, particularly in lane-keeping heightened self-regulation under dual-task demands.

This style-specific pattern is consistent with Zhang et al.(2024), who found that conservative drivers displayed better takeover control in autonomous vehicles under distraction, while aggressive drivers had longer reaction times and lower braking intensity in urgent situations Zhang et al. (2024); Wei et al. (2025).

The differentiated effects of MPDD on lateral and longitudinal control based on driving style highlight the potential for personalized safety interventions. Advanced driver-monitoring systems (DMS) could improve risk prediction by incorporating behavioral style metrics (e.g., headway, speed variability) alongside distraction detection."

## References


Andrikopoulou, E., Spyropoulou, I., 2025. Mobile phone conversation during nighttime driving: Effects on driving behavior. Traffic Injury Prevention 26, 42–51.

Antin, J.F., Lee, S., Perez, M.A., Dingus, T.A., Hankey, J.M., Brach, A., 2019. Second strategic highway research program naturalistic driving study methods. Safety Science 119, 2–10.

Austers, I., Muzikante, I., Cinks, R., 2025. Driving simulator study of text messaging and phone conversations: Effects of messages' valence, drivers' values and self-reported driving behaviors. Heliyon 11.

Basacik, D., Reed, N., Robbins, R., et al., 2011. Smartphone use while driving: a simulator study. Ihs.

Caird, J.K., Johnston, K.A., Willness, C.R., Asbridge, M., Steel, P., 2014. A meta-analysis of the effects of texting on driving. Accident Analysis & Prevention 71, 311–318.

Caird, J.K., Simmons, S.M., Wiley, K., Johnston, K.A., Horrey, W.J., 2018. Does talking on a cell phone, with a passenger, or dialing affect driving performance? an updated systematic review and meta-analysis of experimental studies. Human factors 60, 101–133.

Choudhary, P., Velaga, N.R., 2017. Modelling driver distraction effects due to mobile phone use on reaction time. Transportation Research Part C: Emerging Technologies 77, 351–365.

Choudhary, P., Velaga, N.R., 2019. Effects of phone use on driving performance: A comparative analysis of young and professional drivers. Safety science 111, 179–187.

Chouhan, R., Dhamaniya, A., Rao, A.M., Gupta, K., 2025. Quantifying the relationship between auditory distractions, reaction time, and crash probability. IATSS Research 49, 60–71.

Dingus, T.A., Guo, F., Lee, S., Antin, J.F., Perez, M., Buchanan-King, M., Hankey, J., 2016. Driver crash risk factors and prevalence evaluation using naturalistic driving data. Proceedings of the National Academy of Sciences 113, 2636–2641.

Doshi, A., Trivedi, M.M., 2010. Examining the impact of driving style on the predictability and responsiveness of the driver: Real-world and simulator analysis, in: 2010 IEEE Intelligent Vehicles Symposium, IEEE. pp. 232–237.

Eboli, L., Mazzulla, G., Pungillo, G., 2017. The influence of physical and emotional factors on driving style of car drivers: A survey design. Travel Behaviour and Society 7, 43–51.

Emo, A.K., Matthews, G., Funke, G.J., 2016. The slow and the furious: Anger, stress and risky passing in simulated traffic congestion. Transportation research part F: traffic psychology and behaviour 42, 1–14.

French, D.J., West, R.J., Elander, J., Wilding, J.M., 1993. Decision-making style, driving style, and self-reported involvement in road traffic accidents. Ergonomics 36, 627–644.

Gariazzo, C., Stafoggia, M., Bruzzone, S., Pelliccioni, A., Forastiere, F., 2018. Association between mobile phone traffic volume and road crash fatalities: A population-based case-crossover study. Accident Analysis & Prevention 115, 25–33.

Giot, C., Hay, M., Chesneau, C., Pigeon, E., Bonargent, T., Beaufils, M., Chastan, N., Perrier, J., Pasquier, F., Polvent, S., et al., 2022. Towards a new approach to detect sleepiness: Validation of the objective sleepiness scale under simulated driving conditions. Transportation research part F: traffic psychology and behaviour 90, 109–119.

Hartman, R.L., Brown, T.L., Milavetz, G., Spurgin, A., Pierce, R.S., Gorelick, D.A., Gaffney, G., Huestis, M.A., 2015. Cannabis effects on driving lateral control with and without alcohol. Drug and alcohol dependence 154, 25–37.

Higgs, B., Abbas, M., 2014. Segmentation and clustering of car-following behavior: Recognition of driving patterns. IEEE Transactions on Intelligent Transportation Systems 16, 81–90.

Ishibashi, M., Okuwa, M., Doi, S., Akamatsu, M., 2007. Indices for characterizing driving style and their relevance to car following behavior, in: SICE Annual Conference 2007, IEEE. pp. 1132–1137.

Klauer, S.G., Guo, F., Simons-Morton, B.G., Ouimet, M.C., Lee, S.E., Dingus, T.A., 2014. Distracted driving and risk of road crashes among novice and experienced drivers. New England journal of medicine 370, 54–59.

Li, G., Lai, W., Sui, X., Li, X., Qu, X., Zhang, T., Li, Y., 2020a. Influence of traffic congestion on driver behavior in post-congestion driving. Accident Analysis & Prevention 141, 105508.

Li, S., Zhang, T., Zhang, W., Liu, N., Lyu, G., 2020b. Effects of speech-based intervention with positive comments on reduction of driver's anger state and perceived workload, and improvement of driving performance. Applied Ergonomics 86, 103098.

McEvoy, S.P., Stevenson, M.R., McCartt, A.T., Woodward, M., Haworth, C., Palamara, P., Cercarelli, R., 2005. Role of mobile phones in motor vehicle crashes resulting in hospital attendance: a case-crossover study. Bmj 331, 428.

Mohammadnazar, A., Arvin, R., Khattak, A.J., 2021. Classifying travelers' driving style using basic safety messages generated by connected vehicles: Application of unsupervised machine learning. Transportation research part C: emerging technologies 122, 102917.

National Highway Traffic Safety Administration (NHTSA), 2019. 2018 fatal motor vehicle crashes: Overview. URL: `https://crashstats.nhtsa.dot.gov/Api/Public/ViewPublication/812961`. summary report based on the Fatality Analysis Reporting System (FARS).

Okati-Aliabad, H., Hashemi Habybabady, R., Sabouri, M., Mohammadi, M., 2024. Different types of mobile phone use while driving and influencing factors on intention and behavior: Insights from an expanded theory of planned behavior. PLoS one 19, e0300158.

Organization, W.H., 2015. Global status report on road safety 2015. World Health Organization.

Ortega, C.A.C., Mariscal, M.A., Boulagouas, W., Herrera, S., Espinosa, J.M., García-Herrero, S., 2021. Effects of mobile phone use on driving performance: an experimental study of workload and traffic violations. International journal of environmental research and public health 18, 7101.

Peng, Y., Song, G., Guo, M., Wu, L., Yu, L., 2023. Investigating the impact of environmental and temporal features on mobile phone distracted driving behavior using phone use data. Accident Analysis & Prevention 180, 106925.

Perez, J., Hyun, K., Boni, J.A., 2024. Use of smartphone apps while driving: variations on driving performances and perceived risks. Accident Analysis & Prevention 198, 107474.

Qi, G., Du, Y., Wu, J., Xu, M., 2015. Leveraging longitudinal driving behaviour data with data mining techniques for driving style analysis. IET intelligent transport systems 9, 792–801.

Rumschlag, G., Palumbo, T., Martin, A., Head, D., George, R., Commissaris, R.L., 2015. The effects of texting on driving performance in a driving simulator: The influence of driver age. Accident Analysis & Prevention 74, 145–149.

Shi, J., Xiao, Y., Atchley, P., 2016. Analysis of factors affecting drivers' choice to engage with a mobile phone while driving in beijing. Transportation research part F: traffic psychology and behaviour 37, 1–9.

Stephens, A.N., Groeger, J.A., 2014. Following slower drivers: Lead driver status moderates driver's anger and behavioural responses and exonerates culpability. Transportation research part F: traffic psychology and behaviour 22, 140–149.

Strayer, D.L., Cooper, J.M., Turrill, R.A., Coleman, J.M., Hopman, M.J., Atchley, D.L., 2015. Measuring Cognitive Distraction in the Automobile III: A Comparison of Ten 2015 In-Vehicle Information Systems. Technical Report. AAA Foundation for Traffic Safety. Washington, D.C. URL: `https://appliedcognition.psych.utah.edu/publications/distraction_auto_15.pdf`. accessed: 2025-07-18.

Wei, T., Zhu, T., Zhang, M., 2025. Impact of driving style on rear-end crash risk in leading vehicle emergency braking situations. Proceedings of the Institution of Mechanical Engineers, Part D: Journal of Automobile Engineering , 09544070251323635.

Yan, Y., Zhong, S., Tian, J., Song, L., 2022. Driving distraction at night: The impact of cell phone use on driving behaviors among young drivers. Transportation research part F: traffic psychology and behaviour 91, 401–413.

Yang, L., Ma, R., Zhang, H.M., Guan, W., Jiang, S., 2018. Driving behavior recognition using eeg data from a simulated car-following experiment. Accident Analysis & Prevention 116, 30–40.

Zhang, Y., Ma, Q., Qu, J., Zhou, R., 2024. Effects of driving style on takeover performance during automated driving: Under the influence of warning system factors. Applied Ergonomics 117, 104229.

Zhu, M., Rudisill, T.M., Rauscher, K.J., Davidov, D.M., Feng, J., 2018. Risk perceptions of cellphone use while driving: results from a delphi survey. International journal of environmental research and public health 15, 1074.
