# Supplementary material for: Analysis of the distraction impact on driving performance across driving styles: A driving simulator study in various speed conditions
Source: PLoS One. 2025 Dec 4;20(12):e0336480. doi: 10.1371/journal.pone.0336480 (PMC12677451; doi:10.1371/journal.pone.0336480)
Supplement: Attachment — Submitted filename: Response to Reviewers.pdf [file pone.0336480.s003.pdf]

Authors' response

PONE-D-25-19007

Analysis of the distraction impact on driving performance across driving styles: A driving simulator study in various speed conditions

Mobina Faqani, Habibollah Nassiri, Mahdi Rezaei, Mohsen Ramezani

The authors sincerely thank the editor for managing the review process and the reviewers for their valuable and constructive feedback. We are pleased that the previous concerns have been resolved. In this round, we have revised the Limitations section to explicitly acknowledge the role of drivers' gaze behaviors in characterizing driving style, as suggested by Reviewer 1, and have added the recommended reference. All revisions are highlighted in blue in the revised manuscript.

Reply to Reviewer #1

C1 Thank you for your rely and revisions. My previous concerns have been solved. However, after reading the revised manuscript, I found that there is a new limiation. Although the authors adopted several metrics (e.g., SDLP, ART, and TIBL) to identify driving style, drivers' gaze behaviors are also important aspects of driving style. Thus, I recommend the authors to clarify that. You can refer to this article: Lane changing maneuver prediction by using driver's spatio-temporal gaze attention inputs for naturalistic driving.

R1 We appreciate the reviewer's positive feedback and are pleased that our revisions have addressed the earlier concerns. We also thank the reviewer for this valuable suggestion. We fully agree that gaze behaviors are an important aspect of driving style. In the present study, driving style was identified using performance-based measures (SDLP, ART, and TIBL); however, we acknowledge that the absence of gaze-related data is a limitation of our work. In line with the reviewer's recommendation, we have clarified this point in the Limitations section and included a reference to the suggested article, Lane changing maneuver prediction by using driver's spatio-temporal gaze attention inputs for naturalistic driving.

"While this study utilized a medium-fidelity driving simulator, the use of high-fidelity simulators in future work may enhance validity. Although driving style in the present study was identified using performance-based metrics such as SDLP, ART, and TIBL, we acknowledge that gaze behaviors also represent an important dimension of driving style. Integrating an eye-tracking system into future experiments will enable a more detailed analysis of drivers' visual scanning patterns, including gaze shifts, blink rates, and fixation durations, which are critical for assessing distraction and driving safety. Moreover, future studies could integrate gaze-based measures alongside performance indicators to provide a more comprehensive characterization of driving style (Li et al. (2024)). Examining the relationship between these visual behaviors and driving performance under various mobile phone usage conditions will provide deeper insights into the mechanisms of driver distraction, ultimately contributing to the development of more effective safety interventions."

References

Li, J., Jiang, T., Liu, H., Sun, Y., Lv, C., Li, Q., Yin, G., Liu, Y., 2024. Lane changing maneuver prediction by using driver's spatio-temporal gaze attention inputs for naturalistic driving. Advanced Engineering Informatics 61, 102529.
